# Effect of dietary selenium and omega-3 fatty acids on muscle composition and quality in broilers

**DOI:** 10.1186/1476-511X-6-29

**Published:** 2007-10-29

**Authors:** Anna Haug, Susanne Eich-Greatorex, Aksel Bernhoft, Jens P Wold, Harald Hetland, Olav A Christophersen, Trine Sogn

**Affiliations:** 1 Department of Animal and Aquacultural Sciences, Norwegian University of Life Sciences, 1432 Aas, Norway; 2 Department of Plant and Environmental Sciences, Norwegian University of Life Sciences, 1432 Aas, Norway; 3 National Veterinary Institute, Oslo, Norway; 4 MATFORSK, Norwegian Food Research Institute, 1430 Aas, Norway

## Abstract

**Background:**

Human health may be improved if dietary intakes of selenium and omega-3 fatty acids are increased. Consumption of broiler meat is increasing, and the meat content of selenium and omega-3 fatty acids are affected by the composition of broiler feed. A two-way analyses of variance was used to study the effect of feed containing omega-3 rich plant oils and selenium enriched yeast on broiler meat composition, antioxidation- and sensory parameters. Four different wheat-based dietary treatments supplemented with 5% rapeseed oil or 4% rapeseed oil plus 1% linseed oil, and either 0.50 mg selenium or 0.84 mg selenium (organic form) per kg diet was fed to newly hatched broilers for 22 days.

**Results:**

The different dietary treatments gave distinct different concentrations of selenium and fatty acids in thigh muscle; one percent linseed oil in the diet increased the concentration of the omega-3 fatty acids 18:3, 20:5 and 22:5, and 0.84 mg selenium per kg diet gave muscle selenium concentration at the same level as is in fish muscle (0.39 mg/kg muscle). The high selenium intake also resulted in increased concentration of the long-chain omega-3 fatty acids EPA (20:5), DPA (22:5) and DHA (22:6), thus it may be speculated if high dietary selenium might have a role in increasing the concentration of EPA, DPA and DHA in tissues after intake of plant oils contning omega-3 fatty acids.

**Conclusion:**

Moderate modifications of broiler feed may give a healthier broiler meat, having increased content of selenium and omega-3 fatty acids. High intakes of selenium (organic form) may increase the concentration of very long-chain omega-3 fatty acids in muscle.

## Background

The consumption of broiler meat is high (about 11.5 kg per person per year in Norway [1]). Broiler meat is popular to eat, and the consumption is increasing. Since 1979 the consumption of poultry has increased about five hundred percent [1]. The meat is lean and protein rich and rich in nutrients. Meat quality, fatty acid composition and concentration of several nutrients depend largely on the diet fed to the birds. The typical broiler diet today is cereal based (wheat, barley or corn) and the added fat is mostly rendered fat and vegetable oils. The natural diet for poultry if living in wild environment consists of seeds, plants, insects etc. The concentration of selenium (Se) in wheat grown in Norway is low (wheat grown at Norwegian University of Life Sciences, Aas, Norway contains less than 20 microgram Se/kg wheat, own results). The commercial broiler feed concentrate is supplemented with inorganic selenium (sodium selenite). There are dual benefits from the Se supplementation of broilers; improved health and performance of the animal and improved product quality for human consumption. The concentration of omega-3 fatty acids in animal products depends strongly on the fatty acid composition of the diet [2-7]. Green leaves have a surplus of the omega-3 fatty acid alpha-linolenic acid (ALA) compared to the omega-6 fatty acid linoleic acid (LA). In most seeds and grain, LA dominates, and the concentration of ALA is low. Some seeds have, however, high levels of ALA; e.g. especially linseed but also rapeseed. A hen living in free environments in nature will have a good balance between leaves and seeds in the diet, and thus getting both omega-3 and -6 fatty acids. The feed used by modern poultry industry, however, is based on grain with a high ratio of omega-6 fatty acids compared to omega-3 fatty acids. This will result in a high concentration of the omega-6 fatty acid arachidonic acid (20:4 n-6) in the meat or egg product, and less eicosapentaenoic acid (EPA, 20:5 n-3) and docosapentaenioc- and docosahexaenoic acids (DPA 22:5 n-3 and DHA 22:6 n-3).

Selenium has important physiologic effects that include functioning at the catalytic centre of proteins [8], enhancement of immune function [9], and reduction of cancer risk [10,11].

The low Se intake in many European countries is of concern [12]. The selenium intake may be increased by several means; increased consumption of fish and seafood, increased selenium concentration in cereals and plants by adding selenium to commercial fertilizers, increased concentration in meat and agricultural products by adding selenium to feed, to add selenium supplements directly to the food during processing or to take selenium pills. As selenium is a scarce mineral on our planet and the average concentration in igneous bedrocks is only 0.05 ppm [13], the best and most sustainable way to increase the selenium concentration in the human diet also has to be considered.

The essential long chain omega-6 fatty LA and omega-3 fatty ALA competes with each other in binding to enzymes and incorporation into membrane lipids [14]. ALA intake is associated with inhibitory effects on the clotting activity of platelets, on their response to thrombin, and on the regulation of arachidonic acid metabolism, whereas LA favours oxidative modification of low density lipoprotein cholesterol and increases platelet response to aggregation [15]. The relative proportion between these fatty acids is therefore of importance. The ratio between omega-6 and omega-3 fatty acids is too high in the western diet [16]. Most vegetable oils (except rapeseed oil and linseed oil), most margarines and meat from monogastric animals fed cereal based concentrate feed contribute to this high omega-6 to omega-3 ratio.

The capacity for conversion of ALA to n-3 very long-chain polyunsaturated fatty acids has been investigated [17,18]. It has been shown that this conversion is not high in humans, and it appears that young women of reproductive age have a greater capacity than men to convert the essential fatty acid ALA to DHA [18]. A high Se intake might be speculated to increase the concentration of EPA, DPA and DHA in the tissues either because of increased synthesis from ALA, or reduction in the rate of degradation of EPA, DPA and DHA. Selenide substitutes for sulphide in proteins and enzymes. It has been shown that mammalian cells contain substantial amounts of selenide [19]. Since FeSe is less soluble than FeS [20,21], reflecting the higher degree of covalency in the iron-selenium than in the iron-sulphur bond, it may be speculated that a higher selenide/sulphide ratio in the sulphide sites of the iron-sulphur clusters will stabilize iron atoms in oxidation number +2, making them more difficult to oxidize, thus leading to reduction of the rate of mitochondrial ROS production and reduction of the rate of oxidative attacks. As the rate of peroxidation of polyunsaturated fatty acids depends on the number of double bounds per molecule, the fatty acids EPA, DPA and DHA will be peroxidized faster than the fatty acids with fewer double bounds.

The consumer knows that to eat fish is healthy, but still the fish consumption is not increasing. It has also been predicted that it may be a shortage on fish and fish oil in the future. If broiler meat is produced to be as healthy as possible, with a favourable fatty acid composition and a selenium concentration in broiler meat at about the same level as in fish (0,365 – 0,481 mg/kg in different fish species; turbot, tuna, swordfish, wolfish [22]), intake of broiler meat may improve the average human diet.

The purpose of this investigation was to study how diets containing different amounts of rapeseed oil, linseed oil and selenium rich yeast might affect muscle fatty acid composition and selenium concentration in male broilers, and to produce broiler meat with higher concentrations of omega-3 fatty acids and with a selenium concentration as high as in fish. The effect of high intake of Se (organic form) on muscle concentration of EPA, DPA and DHA was also studied.

## Materials and methods

Sixty newly hatched male broilers (Ross 308, Samvirkekylling, Norway) were used in this experiment that was conducted at The Norwegian University of Life Sciences. Broilers were weighed and assigned randomly to one of four dietary treatments. The experiment lasted for 22 days. The first 10 days the birds from each group were kept in deep littered pens; 75 cm × 150 cm. At day 10 each individual were weighed and 15 birds in each group were placed in separate wire-floored metabolism cages, and kept there from day 10 to day 22. Chicks were raised in an environmentally controlled room. The room temperature was maintained at 32°C from days 0–3, then gradually reduced by 0,5°C per day until day 22. Chicks were exposed to 23 h light and 1 h dark photoperiod days 0–7. The next two weeks they were exposed to 2 × 4 h dark; the dark periods were between 17–21 h and 00–04 h. The birds had free access to food and water. Water troughs and wire-floors were cleaned daily. At the end of day 19 and 22 feed intake and body weight were determined for each individual. Signs of disease or mortality were recorded daily (but in this experiment none of the birds became ill or died).

### Experimental procedures

At day 22 individual blood samples were collected by wing venipuncture into 7 ml tubes containing heparin. One ml of the heparin-blood was frozen immediately for glutathione peroxidase determination. The remaining of the blood samples were centrifuged for 15 minutes at 3,000 × g at 4°C. The plasma was frozen at -20°C in order to determine total antioxidant status. After bleeding, the broilers were sacrificed. The left and right thighs were obtained to determine selenium and fatty acid composition of muscle. Breast muscles were dissected out for sensory quality analyses. The liver and heart were weighed. The muscle samples were stored at -20°C before being analyzed.

### Diet formulation

The four diets were wheat-based (60% wheat) containing soybean meal, fish meal, vitamins and minerals. The diets were formulated by combining different levels of rapeseed oil (RO) and linseed oil (LO) with two different concentrations of selenium enriched yeast; 0.02% or 0.04%. The final Se concentration in the diets with 0.02% Se enriched yeast was 0.50 mg Se/kg diet, and the final concentration in the diets containing 0.04% Se enriched yeast was 0.84 mg Se/kg diet, thus the dietary treatments are named were named: RO+50Se, RO+84Se, LO+50Se and LO+84Se.

The dietary treatment RO+50Se and RO+84Se include rapeseed oil (5%) and rendered fat (5%). Diet LO+50Se and LO+84Se contain 1% linseed oil, 4% rapeseed oil and 5% rendered fat, and 0.02% selenium enriched yeast in LO+50Se, and 0.04% selenium enriched yeast in LO+84Se. Diet compositions are listed in Table 1.

**Table 1:** Diet composition.

Ingredients (%)	RO+50Se	RO+84Se	LO+50Se	LO+84Se
Wheat	60	60	60	60
Soybean meal	20	20	20	20
Fish meal	3	3	3	3
Rapeseed oil	5	5	4	4
Linseed oil	0	0	1	1
Rendered fat	5	5	5	5
Soybean oil	0	0	0	0
Mono calcium phosphate	2	2	2	2
Ground limestone	1,85	1,85	1,85	1,85
Sodim chloride	0,25	0,25	0,25	0,25
DL-Methionine	0,4	0,4	0,4	0,4
L-Lysine	0,3	0,3	0,3	0,3
L-Threonine	0,1	0,1	0,1	0,1
Choline chloride	0,13	0,13	0,13	0,13
Manganoxid	0,00657	0,00657	0,00657	0,00657
Mikromin FKRA 30 *	0,15	0,15	0,15	0,15
Vitamin mixture**	0,08	0,08	0,08	0,08
Se-methionine rich yeast***	0,02	0,04	0,02	0,04

*Supplied (mg/kg of diet): Ca 315, Fe 75, Mn 60, Zn 105, Cu 15, I 0,75,

**Vitamin mixture provided the following per kg diets: retinyl acetate, 3.1 mg; cholecalciferol, 0.07 mg; DL-alpha-tocopheryl acetate, 38 mg; menadione, 2.25 mg; pyridoxine, 3.4 mg, riboflavin,9 mg; Ca-pantothenate, 12.5 mg; biotin, 0.19 mg, thiamine, 1.9 mg; niacin, 37.5 mg; cobalamin, 0.02 mg; folic acid 1.5 mg.

*** Organic selenium Yeast (BioLogics, Ultra Bio-Logics Inc. New O.S.Y. 2000X)

Rapeseed oil: Askim fruktpresse, Norway; 60% oleic acid, 20% linoleic acid and 10% alphalinolenic acid.

Linseed oil: Fedon Lindberg linseed oil, Norway; 26% oleic acid, 21% linoleic acid and 45% alphalinolenic acid.

Rendered animal fat (from ruminants (about 70%) and pigs (about 30%). Fatty acid composition: 26% palmitic acid, 18% stearic acid, 36% oleic acid, 8% linoleic acid. ForTek, Ås, Norway.

Fatty acid composition of diets (g/100 g fatty acids) is shown in Table 2.

**Table 2:** Fatty acid composition of diets, g/100 g fatty acids.

	RO+50Se	RO+84Se	LO+50Se	LO+84Se
C14:0	0,67	0,80	0,87	0,92
C16:0	12,59	13,13	15,07	15,10
C16:1	0,98	1,04	1,16	1,27
C18:0	6,11	6,23	7,42	7,63
C18:1	36,12	38,15	39,91	38,58
C18:2	20,75	20,13	21,74	21,18
C18:3	5,07	5,28	8,69	9,24
18:2/18:3	4,09	3,81	2,50	2,29

All the diets are rich in oleic acid (18:1), varying from about 36 to 40 g oleic acid per 100 g fat in the RO and LO diets. The diets are also rich in linoleic acid (18:2); accounting to about 20 g per 100 g fat in the diet, and alpha-linolenic acid (18:3) accounting to about 5% of the fat in the RO diets, and about 9% of the fat in the LO diets. Thus the ratio between linoleic acid and alpha linolenic acid is much higher in the RO diet than in the LO diet. The palmitic acid (16:0) content is highest in the LO diets.

### Laboratory analyses

#### Fatty acid analyses

Fatty acid composition of thigh muscle, feed and oils were determined by gas chromatography. In muscle, lipid extraction was performed according to Folch [23], on 1 g of thigh muscle tissue. The lipids were resolved in heptane, and methylated as described [24], using both sodium methoxide and methanolic HCl 3N (Supelco, PA, USA). Subsequently, the fatty acid methyl esters were analyzed using a Finnigan Focus gas chromatograph with a 100 m capillary column (CP Sil 88 WCOT, 100-m × 0.25 m m, Chrompack, Middelburg, Netherland). Injector and detector temperatures were 250° and 255°C, respectively and oven temperature was 70° initially. After 4 minutes the temperature was increased by 13°C/min to 175°, held there for 27 minutes, programmed at 4°C/min to 215°C, held there for 31 minutes and programmed at 10°C/min to a final temperature of 225°C. The flow rate of the carrier gas, helium, was 1.62 ml/minute, a 1:34 split mode and a flame-ionization detector. Fatty acid peaks determined by gas chromatograph were then used to calculate the amount of fatty acids (g/100 g fat) by theoretical response factors [25]. Standard fatty acids of known composition were run to identify the fatty acids in the samples. Muscle control samples was extracted, methylated and analysed by every 10^th ^sample. The coefficient of variation (CV) for the individual fatty acids was found to vary between 4 and 6.5%.

#### Selenium

Selenium concentration in the diets and leg muscle of each broiler were analyzed by atomic absorption spectrometry with a hydride generator system [26] using Varian SpectrAA-30 with a VGA – 76 vapour generation accessory. Before analysis, each sample was prepared by oxidative digestion in a mixed solution with concentrated nitric and perchloric acids, using an automated system with Tecator 1012 Controller and 1016 Digester heating unit. The method is accredited (NS-EN ISO/IEC 17025). A quality control system using regular analyses of a blood standard (proficiency tested pig blood 1992) with value 0.20 ± 0.02 μg/g was adopted as reference material [27]. The detection limit was 0.01 μg/g.

Whole blood was analyzed for ***glutathione peroxidase (Gpx) ***according to [28], where Gpx catalyses oxidation of glutathione and the oxidized glutathione is reduced back to reduced form at the same time as NADPH is oxidized to NADP, giving a decrease in absorbance at 340 nm.

##### Total antioxidant status (TAS)

was based on the generation of the ABTS (2,2-Azino-di-(3-ethylbenzthiazoline sulphonate) radical cation from the interaction of methemoglobin, ABTS and hydrogen peroxide. TAS was measured using Randox kit supplied by Randox Laboratories Ltd. UK.

##### 2-Thiobarbituric Acid (TBA) Assay

Lipid oxidation in this experiment was determined by the TBA method described by Sørensen and Jørgensen [29]. Briefly, 10.0-grams of grinded broiler muscle from right thigh were placed in MSE-homogenization tubes, and blended for 30 s with 30 ml 7,5% TCA (Merck) containing 0,1% propylgallat (Merck) and 0,1% EDTA (Merck). The slurry was filtered through a Whatman No. 1 filter. Five ml of the filtrate and 5 ml 0,02 M 2-thiobarbituric acid (Sigma Chemical Co., St. Louis, Mo) were mixed, heated for 35 minutes in boiling water bath, and the colour was measured by a Pharma, Biotech Ultraspec 3000, Cambridge, UK, at a wavelength of 532 nm. Thiobarbituric acid reactive substances, expressed as milligrams of malondialdehyde per kilogram of meat, were calculated from the standard curve of TEP (1,1,3,3 -tetra-ethoxypropane).

##### Front-Face Fluorescence Spectroscopy (FFF)

FFF measurements were performed with an optical bench system described by Wold and Mielnik [30] and the spectra can be used as an indicator of lipid oxidation [30]. In general, the higher fluorescence intensity around 470 nm, the more oxidised is the sample. Fluorescence emission spectra were measured directly on the illuminated samples of minced broiler thigh plus leg meat. Round, flat, black, plastic cuvettes (diameter 5 cm) were filled with sample, and the top was flattened to a smooth surface. The samples were exposed to 382 nm excitation light and emitted fluorescence light was measured from 410 to 750 nm.  The spectra were collected by an imaging spectrograph (Acton SP-150, Acton Research Corp., Acton, MA) connected to a sensitive charge coupled device (CCD camera) (Roper Scientific NTE/CCD-1340/400-EMB, Roper Scientific, Trenton, NJ). The samples were illuminated for 4 s, rotated approximately 90°, and illuminated again, giving two readings for each sample. The readings were averaged prior to data handling.

#### Sensory Analysis

A professional sensory panel with 11 assessors evaluated the breast filet samples in a descriptive test according to an accredited method [31]. The breast filets had been stored individually wrapped in aluminium folio at -20°C for five months.

Prior to the analyses, the panellists were trained on extra sample that had been stored in -20°C for five months, before they started on real samples at the days of analysis. The vocabulary included odour, flavour and colour. The samples were prepared by dividing all the breast filets into two halves, and each peace were vacuum packed in individual plastic bags, and immersed in water bath at 80°C for 30 minutes. Then the pieces were immediate distributed to the panellists, one to each assessor. The samples were marked with random three digit numbers and presented to the panellists in randomized order. Scores were recorded on a continuous, linear scale from 1 (no intensity) to 9 (distinct intensity) with a Compusense Five software (v.4.2, Compusense Inc., Guelph, ON, Canada).

#### Statistical methods

Two-way analyses of variance were performed using the GLM procedure of SAS software (SAS Institute Inc., Cary, NC, USA). Significant differences between treatments were determined by using the Ryan-Einot-Gabriel-Welch *F*-test. Square root of MSE (RSD) was used as a measure of random variation. The sensory results were analyzed by ANOVA analysis of variance and Tukeys test. The fluorescence measurements resulted in spectra covering the range 410–750 nm. Systematic variation from these spectra was extracted by principal component analysis (PCA). The PCA decomposes the spectral variables into a few so-called principal components, which reflect the main spectral variation. The second PC was used as an indicator of lipid oxidation.

Selenium concentration in liver, muscle and excreta was determined at the National Veterinary Institute, Norway. Whole blood was analyzed for glutathione peroxidase (Gpx) and total antioxidant status (TAS) was determined in the plasma at The Norwegian University of Life Sciences. TBA, FFF and sensory analyses were done at MATFORSK, Aas, Norway.

## Results

The results of dietary treatment with different fat source; rapeseed oil (RO) or rapeseed oil plus linseed oil (LO), and with different selenium concentration; 0.50 mg/kg or 0.84 mg/kg diet calculated by two-way analysis of variance are shown in Table 3. Final body weight at day 22 was a little higher in the broilers in the RO dietary treatment, compared to the LO dietary treatment. Feed intake was also a little higher (not significant) in the rapeseed group and feed conversion were not different among the dietary treatments (results not shown).

**Table 3:** Final body weight (FBW), liver weight, heart weight, glutathione peroxidase activity (GPX, U/ml), total antioxidant status (TAS, mmol/l), mg selenium/kg leg muscle, thiobarbituric acid reactive substances (TBA) mg/kg leg muscle and front face fluorescence spectrometry (FFF) and thigh muscle fatty acid composition (g/100 g fatty acid) for broilers fed diets based on wheat with different fat source; rapeseed oil (RO) or rapeseed oil plus linseed oil (LO), and with different selenium concentration: 0.50 mg Se/kg or 0.84 mg Se/kg diet.

	**Fat source**		**Selenium**		**P (Fat source)**	**P (Selenium)**	P (Fat source*Selenium)	**RSD**
	**RO**	**LO**	**50Se**	**84Se**				
FBW	783	749	775	756	0,04	NS	NS	64,12
Liver g	26,1	24,8	25,8	25	NS	NS	0,003	2,63
Heart g	7,24	6,51	7,1	6,7	0,007	NS	NS	1,02
Se mg/kg muscle	0,33	0,34	0,28	0,39	<0,0001	<0,0001	<0,0001	0,012
Gpx U/ml	14,4	14,5	12,5	16,3	NS	<0,0001	NS	2,29
TAS mmol/l	0,79	0,71	0,77	0,74	NS	NS	NS	0,186
FFF	76	410	245	224	NS	NS	NS	1597
TBA ug/g muscle	0,35	0,3	0,31	0,33	0,012	NS	NS	0,075
*Thigh muscle fatty acids, g/100 g fat:*								
C14:0	0,38	0,41	0,4	0,39	NS	NS	0,018	0,1
C14:1	1,98	2,01	1,91	2,14	NS	0,013	NS	0,35
C16:0	14,44	14,89	14,5	14,83	0,057	NS	NS	0,91
C16:1	1,07	1,27	1,21	1,13	0,019	NS	0,043	0,32
C18:0	11,3	11,3	11,1	11,5	NS	NS	0,017	1,19
C18:1	23,1	22,6	23,5	22,3	NS	NS	NS	2,52
C18:2	16,4	15,5	16	16	0,001	NS	0,019	1,03
C18:3	1,71	2,59	2,2	2,1	<.0001	NS	NS	0,48
C20:4	5,09	4,22	4,56	4,75	0,0002	NS	NS	0,84
C20:5	0,88	1,41	1,07	1,22	<0,0001	0,016	NS	0,23
C22:5	2,24	2,73	2,31	2,66	0,0002	0,006	0,059	0,482
C22:6	3,32	3,27	3,11	3,48	NS	0,035	NS	0,673
18:2/18:3	9,97	6,34	8,1	8,3	<.0001	NS	NS	1,94
20:4/20:5	5,94	3,05	4,72	4,26	<0,0001	NS	NS	0,996

Heart weight was highest for the RO dietary treatment, whereas it was an interaction between fat source and selenium; the product of fat source*selenium significantly affected liver weight.

The Se concentration in broiler thigh muscle was lowest in the treatment group 50Se, and highest in the treatment group, 84Se. The Se concentration in muscle was also slightly affected by the fat source, and muscle Se was highest in the LO treatment compared to RO dietary treatment (0.34 and 0.33 microgram Se/g muscle, respectively). The product (fat source*selenium) also showed significance, showing interaction between the dietary treatments.

Glutathione peroxidase (Gpx) was lowest in the treatment group 50Se, and highest in the treatment group, 84Se.

Total antioxidant status (TAS) and Front-Face Fluorescence Spectroscopy (FFF) was not affected by the dietary treatments. Thiobarbituric acid reactive substances (TBA), expressed as milligrams of malondialdehyde per kilogram of meat was affected by fat source, being highest in the RO dietary treatment.

The fatty acid concentrations in thigh muscle were affected by dietary treatments. The LO treatment resulted in higher concentrations of the polyunsaturated fatty acids 18:3, 20:5 and 22:5, and the monounsaturated fatty acid 16:1. The concentrations of 18:2 and 20:4 in muscle were lower in the LO dietary treatment groups. The ratio between 18:2 and 18:3 and the ratio between 20:4 and 20:5 was also lower in the LO dietary treatment group.

The dietary selenium concentration affected the fatty acid concentration. The concentration of the very long chain polyunsaturated fatty acids 20:5, 22:5 and 22:6 were highest in the high Se (84Se) treatment group. The concentration in muscle of the monounsaturated fatty acid 14:1 was also increased by high dietary Se.

The **sensory analysis **showed no significant differences among the groups on the sensory quality of cooked breast muscle (Table 4), when tested for acidulous odour, metallic odour, cabbage odour, nut odour and rancid odour, and acidulous, sweet, metallic, bitter, cabbage, nut and rancid flavour. In this experiment, the broiler meat was not affected by the dietary treatments regarding the chosen sensory traits, and in the descriptive test there were no significant differences between the samples for any of the 12 parameters included in the test. All the samples had clear intensity of metal-smell, metal-taste, and bitter taste. This may be caused by small rests of blood on the samples.

**Table 4:** Sensory quality of cooked broiler breast meat.

		RO+50Se	RO+84Se	LO+50Se	LO+84Se
Odour					
	Acidulous	2,52	2,71	2,85	2,99
	Metallic	4,44	4,35	4,33	4,37
	Cabbage	2,19	2,09	2,01	2,01
	Nut	1,00	1,06	1,05	1,06
	Rancid	2,00	1,68	1,63	1,54
Flavour					
	Acidulous	2,62	2,71	2,89	2,89
	Sweet	2,75	2,88	2,82	2,72
	Metallic	4,64	4,49	4,66	4,73
	Bitterness	4,48	4,27	4,11	4,13
	Cabbage	2,57	2,27	2,64	2,31
	Nut	1,13	1,19	1,16	1,12
	Rancid	2,82	2,75	2,30	2,43

Sensory traits were evaluated from 1 to 9, where 1 is no intensity and 9 is high intensity.

## Discussion

Dietary treatments with rapeseed oil (RO), linseed oil (LO) and two levels of selenium enriched yeast (50Se and 84Se) gave major effects on muscle Se concentration and fatty acid concentration in broiler leg muscle, but small effects on final body weight and thiobarbituric acid reactive substances, and no effects on total antioxidant status, front-face fluorescence spectroscopy and sensory quality. In the present study, selenium enriched yeast (0.04% in the diet) resulted in increased concentrations of the very long chain fatty acids EPA, DPA and DHA in broiler thigh muscle.

Final body weight and heart weight was affected by fat source, showing higher body and heart weight by the RO treatment than in the LO treatment groups. This might be caused by a firmer pellet quality in the RO feed (observed visually) than in the LO groups, possibly because the more polyunsaturated linseed oil affects the melting point giving a more "floury" feed that is more difficult to eat for the bird. Higher organ weight is expected in birds with higher body weight. As expected, liver weight was higher, but did not reach significance.

Selenium concentration in raw thigh muscle was significantly increased (33% increase) in the diet with most Se-enriched yeast (84Se) compared to the diet with less Se-enriched yeast (50Se). This increase is in accordance to several studies showing that organic forms of Se in the diet gives increased tissue concentrations of Se [32-34]. The yeast mainly contains Se in the form of selenomethionine, which can't be synthesized by poultry and mammals [34]. Selenomethionine is absorbed as an amino acids in the intestine, and in the tissues it is competing with methoinine in building in to proteins [33]. Selenomethionine can also be converted to selenocysteine and incorporated into e.g. Gpx [34].

The effect of fat source on Se concentration in muscle and the interaction of fat source and selenium are interesting. The average Se muscle concentration from RO and LO dietary treatment is not much different in numerical value (0.33 mg/kg and 0.34 mg/kg in RO and LO treatment), but highly significant from each other. It might be speculated that the LO diet might have a better absorption or utilization of Se because of the more "floury" pellet quality. Another suggestion may be that long chain polyunsaturated fatty acids results in an increased concentration of Se-containing proteins in muscle tissue. More oxidizing conditions (e.g. higher rate of ROS production) is known to lead to induction of Se-containing antioxidative enzymes such as Gpx 1, Gpx 4, selenoprotein W or thioredoxin reductase both in skeletal muscle and other organs [35-37]. It might be speculated that the increased dietary intake of polyunsaturated fatty acids leads to induction of Se-containing antioxidative enzymes in broiler skeletal muscle.

There were no differences among the groups in plasma total antioxidant status, front-face fluorescence spectroscopy and sensory evaluation on meat stored frozen for 5 months. These parameters are indicators of oxidation, and although selenium takes part in antioxidant enzymes, and linseed oil is highly polyunsaturated and prone to oxidation, no differences among the groups were shown in this study. The selenium intake was high in both groups, and therefore saturation of Gpx isozymes and selenoprotein P must expected in this study [38,39]. The birds were young and fed a diet rich in essential nutrients, giving healthy birds with a good antioxidant defence, and thus explaining the lack of differences in antioxidative parameters among the treatment groups.

The fatty acid composition of the meat was affected by the dietary fat source, and this has been reported by others [32,40-43]. The omega-3 fatty acid ALA is highest in the LO diet, and it is significantly higher in the muscle of LO treated birds. The other omega-3 fatty acids 20:5 (EPA) and 22:5 (DPA) are also increased following linseed oil intake, and this may be a result of elongation and desaturation of ALA to EPA and DPA, as has been reported in broilers by others [32]. The decrease in the broiler muscle omega-6 fatty acids LA and arachidonic acid in the LO treatment groups may be explained by a competition between LA and ALA in incorporation into tissue membranes and desaturases and elongases of the 18 carbon fatty acids to 20 carbon fatty acids. The ratio between LA and ALA (18:2 n-6 and 18:3 n-3) and between arachidonic acid and EPA (20:4 n-6 and 20:5 n-3) is significantly much lower following the LO treatment, giving a more favourable omega-3 status in the LO-broilers.

In the western diet the intake of omega-3 fatty acids is too low resulting in increased clotting of platelets, inflammation, cardiac diseases and cancer [16]. Meat that is rich in omega-3 fatty acids might therefore have a health advantage compared to the regular meat product. But also in the RO treatment groups the omega-3 fatty acid concentrations are high and favourable. Therefore the meat from the RO treatment group is a better alternative than the regular broiler that is offered in the supermarkets in Norway, because they have been given a cereal based feed without much omega-3 fatty acids (the fatty acid composition in chicken is reported to be 18:2;12.2%, 18:3; 0,9%, 20:4; 0,5%, 20:5; 0,3%. [44].

The concentration of 16:1 is higher in the LO diets, and this is reflected in the muscle 16:1 in the LO treated groups, and there is an interaction between fat and selenium in this parameter.

In the present study, dietary selenium affects fatty acid concentrations; the highest selenium intake increases the muscle concentrations of the very long chain polyunsaturated fatty acids EPA, DPA and DHA compared to the 50Se treatment. Also the monounsaturated 14:1 fatty acid is increased by high dietary Se. The very long chain fatty acids are produced by delta-4, delta-5 and delta-6 desaturases and elongases. In this study we could not determine where in the fatty acid C-chain the double was located, and thus we do not know it the 14:1 fatty acid is 14:1 n-5 (produced by delta-9 desaturase), or if it is produced by other possible desaturases. The 16:1 fatty acid is commonly reported to be 16:1 n-7, thus produced by delta-9 desaturase, and in the present study this fatty acid is not increased by high dietary selenium. It may be speculated that the activity of delta-4, 5 or 6 desaturases or elongase may be increased by high Se intakes in broiler or that high Se intakes may lead to reduction of the rate of degradation of these fatty acids by processes of peroxidation.

It may be possible that the Se intake needed for saturation of some of the antioxidative selenoenzymes in muscle cells (Gpx 1, Gpx 4, thioredoxin reductase and selenoprotein W) is higher than needed for saturation of selenoenzymes in blood plasma and blood cells. Higher level of antioxidant enzymes will lead to reduction of the rate of lipid peroxidation, which means reduction of the rate of EPA, DPA and DHA degradation by peroxidation.

Another possibility may be that the oxidation of EPA, DPA and DHA in mitochondria may be reduced as a consequence of selenide replacing sulphide in mitochondrial iron-sulphur enzymes. This might change the kinetic properties of these enzymes, since a change of the Se^--^/S^-- ^ratio at the sulphide positions must be expected to affect the standard redox potential for the Fe^++^/Fe^+++ ^equilibrium. Fe^++ ^is bound more strongly to selenide than to sulphide ions as illustrated by the much lower solubility product of FeSe (10^-26^) [20] compared with FeS (4 × 10^-19^) [21]. A higher selenide/sulphide ratio in the iron- sulphur clusters would therefore be expected to enhance the stability of ferrous iron, i.e. to enhance the standard redox potential for the Fe^++^/Fe^+++ ^equilibrium. It might be speculated that this could lead to enhancement of the rate of mitochondrial NADH oxidation, other factors being equal. This might in turn enhance the rate of electron flow from NADH to cytochrome *c *oxidase, leading to enhancement of the rate of O_2 _reduction by the latter enzyme. Enhancement of the rate of O_2 _reduction might in turn lead to reduction of the intracellular O_2 _partial pressure in most organs, and to reduction of the rates of reactive oxygen species (ROS) production and lipid peroxidation.

The rate of peroxidation of polyunsaturated fatty acid groups depends on the number of double bonds per fatty acid molecule [45]. The number of double bonds is especially high in EPA, DPA and DHA, making these fatty acids particularly vulnerable to peroxidative attack. More oxidizing conditions (e.g. higher rate of ROS production) must therefore be expected to affect the rates of EPA, DPA and DHA peroxidation more strongly than it affects the rates of peroxidation of fatty acids with a smaller number of double bonds, such as LA.

Iron is a very important catalyst of lipid peroxidation reactions [46,47]. It might be speculated that a higher selenide/sulphur ratio in iron sulphur proteins also might affect the concentration of iron that is bound to small molecules and functions as a catalyst of peroxidation reactions. This is another putative mechanism, by which a higher selenide/sulphide ratio in mitochondrial enzymes might lead to reduction of the rate of lipid peroxidation reactions, with this effect being especially pronounced for those fatty acids that have the largest number of double bonds.

An increase in the very long chain omega-3 fatty acids EPA, DPA and DHA in muscle by a diet rich in Se is highly interesting. Pending further investigations, it is necessary to keep all possibilities open regarding the possible mechanisms. In humans the concentration of these fatty acids are also dependent both on the rates of intake plus synthesis and degradation. The conversion of ALA to EPA, DPA and DHA is reported to be low in humans [17]. If poor selenium status should lead to enhancement of the rate of EPA, DPA and DHA degradation or reduced synthesis, this will interact synergistically with low dietary intake of these fatty acids. An increased intake of Se in the diet might thus have practical implications by increasing the level of these valuable fatty acids. A health benefit of high Se intakes on disorders related to lack of long chain omega-3 fatty acid might be suggested.

## Conclusion

In conclusion, some moderate modifications of broiler feed by supplementation of rapeseed and linseed oil and adding Se-enriched yeast to the feed, gives broiler muscle enriched in selenium and omega-3 fatty acids. Consumption of such a meat product may be beneficial to human health. The increased concentration of long-chain omega-3 fatty acids observed in muscle of broilers that has been fed a diet containing 0.84 mg Se in organic form per kg diet, is interesting, and deserves further investigation.

## Authors' contributions

A. Haug; major contributor; in planning, experimental work, analysis and publication of results. S. Eich-Greatorex; contributed in planning of experiment and in the practical work in the finishing of experiment, with taking blood samples and removing and handling organs for further analysis. A. Bernhoft; contributed in planning of experiment and in selenium determination. Harald Hetland; contributed in planning of the experiment and in formulation of diets and the upset of the broiler experiment. Jens P Wold; contributed in planning and analysing. Olav A. Christophersen contributed in planning of the experiment and in discussion of results. Trine Sogn; major contributor in planning of the experiment and in providing funding for the study.
